# A Series of Questions Pertaining to the Curriculum of the Dental Student

**Published:** 1885-11

**Authors:** 


### Bibliographical.

A Series of Questions Pertaining to the Curriculum of the Dental Student.—Embracing Dental Histology, Dental Pathology, Dental Surgery, Dental Prosthesis, Dental Matallurgy, Dental Materia Medica and Therapeutics, Anatomy, Physiology and Chemistry. By Ferdinand J S. Gorgas, A M., M. D., D. D. S., University of Maryland. Publishers: W. K. Boyle & Son., Cor. Baltimore and St. Paul Street, Baltimore, Md. 1885. Price $1.50.

This work comprises leading questions on all the branches belonging to the course of study pursued by the dental student, and its object is to facilitate the study of dental science and its collateral sciences.

Some years ago the author published a small work embracing in an abridged form, questions on Dental Science, and the favor with which it was received by the students of his class, was such as to rapidly exhaust the limited edition, and induced him to prepare, when opportunity offered, a more complete series of questions embracing the entire curriculum of the dental student, and even that of the medical student so far as related to Anatomy, Physiology and Chemistry.

The present work, therefore, is the result of such an intention, and is presented with the hope that it may accomplish the object for which it has been prepared.

